# Supplementary material for: Overprescribing among older people near end of life in Ireland: Evidence of prevalence and determinants from The Irish Longitudinal Study on Ageing (TILDA)
Source: PLoS One. 2022 Nov 30;17(11):e0278127. doi: 10.1371/journal.pone.0278127 (PMC9710761; doi:10.1371/journal.pone.0278127)
Supplement: S1 File — (DOCX) [file pone.0278127.s001.docx]

**Supplementary Material**

This supplementary material file contains the following information:

1. Additional detail on the mortality index methodology
2. Classification of potentially inadequate and inappropriate medications
3. How the analytic sample was reached
4. Sensitivity analysis for imputed values
5. Secondary analysis of specific medications and association with gender

**1) Mortality Index Method**

We developed and validated a mortality index in TILDA) based on previously published mortality indices developed by Lee and colleagues (2006)^1^ in the United States (Health Retirement Study; HRS) and Kobayashi and colleagues (2017)^2^ in the United Kingdom (English Longitudinal Study of Ageing; ELSA). We followed the same statistical methods employed in those indices in our data; the derived index had equivalent predictive performance: AUC= 0.78. The development of our full TILDA mortality risk index is discussed in detail elsewhere^3^.

Our final 14-predictor model assigned risk points for: male (1pt); age (65–69: 2pts; 70–74: 4 pts; 75–79: 4pts; 80–84: 6pts; 85 + : 7pts); heart attack (1pt); cancer (3pts); smoked past age 30 (2pts); difficulty walking 100 m (2pts); difficulty using the toilet (3pts); difficulty lifting 10lbs (1pts); poor self-reported health (1pt); and hospital admission in previous year (1pt). Within the Irish 14-predictor mortality index, the lowest score available is 0 points and highest is 22 points. We mapped the two-year mortality of participants against their mortality index score and there is a spike at 11 points from <10% to 25%< two-year mortality. Participants were therefore included in our analytic dataset in this study at the first Wave at which they have 11+ points on the mortality index score.

**Supplementary Materials Figure 1 Two-year mortality prediction**

**2) Medications and Drug Classes**

**Supplementary Material Table 1 Medications and drug classes categorised as questionable or often inadequate by Morin et al.**

| **Questionable** | **Often inadequate** |
| --- | --- |
| Drugs for acid-related disorders, excl. PPI (A02) | Vitamin D (A11CC) |
| Oral antidiabetics, excluding metformin (A10B) | Calcium supplement (A12A) |
| Vitamin K antagonists (B01AA) | Cardiac stimulants other than glycosides (C01C) |
| Unfractionated heparin (B01AB01) | Antihypertensives, excluding α-blockers (C02) |
| Platelet aggregation inhibitors, incl. low-dose aspirin (B01AC) | Peripheral vasodilators (C04) |
| Novel oral anticoagulants (B01AE, B01AF) | Lipid-modifying agents (C10) |
| Other anticoagulants (B01AD, B01AX) | Immunostimulants (L03A) |
| Antianemic preparations (B03) | Bisphosphonates (M05BA) |
| Blood products (B05A) | Other osteoporosis drugs (M05B) |
| Cardiac glycosides (C01A) | Antidementia drugs (N06D) |
| Alpha-blocker antihypertensives (C02CA, C02LE) |  |
| Low-ceiling diuretics, thiazides & non-thiazides (C03A, C03B) |  |
| Potassium-sparing agents, excl. spironolactone (C03D) |  |
| Non-selective beta-blockers (C07AA) |  |
| Calcium channel blockers (C08) |  |
| Angiotensin-converting-enzyme inhibitors (C09A, C09B) |  |
| Angiotensin II antagonists (C09C, C09D) |  |
| Finasteride (G04CA51) |  |
| Iodine therapy (H03C) |  |
| Antineoplastic drugs (L01) |  |
| Endocrine therapies (L02) |  |
| Immunosuppressants (L04A) |  |
| Anti-gout drugs, excluding allopurinol and colchicine (M04) |  |
| Systemic drugs for obstructive airway diseases (R03C, R03D) |  |

**3) Eligible TILDA Sample**

The TILDA sample across the first four Waves is presented in Supplementary Material Table 2. Of 8,174 participants in Wave 1, 150 (2%) had high mortality risk and were included in the analytic sample. A further 122, 138 and 115 participants were eligible in Waves 2-4 respectively to give an analytic sample with 525 observations.

**Supplementary Material Table 2 TILDA participation, attrition and mortality, Waves 1-4**

|  | Wave 1 | Wave 2 | Wave 3 | Wave 4 | *Analytic sample* |
| --- | --- | --- | --- | --- | --- |
| Participated | 8,174 | 7,282 | 6,619 | 5,942 |  |
| *>>Of whom had high mortality risk* | *150* | *122* | *138* | *115* | *525* |
| No participation, assumed alive |  | 972 | 1,440 | 1,904 |  |
| Deceased (cumulative) |  | 208 | 528 | 789 |  |
| TOTAL | 8,174 | 8,462 | 8,587 | 8,635 |  |

High mortality risk = 11+ points on our mortality risk score.

**4) Sensitivity analyses for imputation**

Below are Supplementary Material Table 3 and Supplementary Material Table 4 that show the regression results without imputed data for potentially inadequate and questionable prescribing.

Regression results for potentially inadequate medications are presented in Supplementary Material Table 3, with statistically significant (p<0.05) associations highlighted bold. There are two such predictors: male (OR 5.67, *p*=.002); and three or more ADLs (OR 3.51, *p*=.03).

**Supplementary Material Table 3 Multivariable regression results for potentially inadequate medications without imputed values.**

| Variables | Odds Ratio | P>z | [95% Conf. | Interval] |
| --- | --- | --- | --- | --- |
| Gender (male) | **5.67** | **<0.01** | **1.87** | **17.15** |
| Education |  |  |  |  |
| *Secondary* | 0.36 | 0.12 | 0.10 | 1.32 |
| *Third/higher* | 0.61 | 0.42 | 0.18 | 2.05 |
| Age group |  |  |  |  |
| *70-74 years* | 1.91 | 0.60 | 0.17 | 21.60 |
| *75-79 years* | 2.88 | 0.37 | 0.28 | 29.20 |
| *80-84 years* | 1.75 | 0.65 | 0.16 | 19.07 |
| *85 years and over* | 6.11 | 0.12 | 0.64 | 58.54 |
| Cancer | 1.14 | 0.81 | 0.38 | 3.43 |
| Any serious heart condition | 0.64 | 0.42 | 0.22 | 1.88 |
| Any heart risk factor(s) | 1.54 | 0.55 | 0.37 | 6.44 |
| More than one serious chronic condition | 1.31 | 0.10 | 0.95 | 1.80 |
| Activities of daily living |  |  |  |  |
| *1 ADL* | 0.33 | 0.32 | 0.04 | 2.98 |
| *2 ADL* | 1.18 | 0.85 | 0.22 | 6.23 |
| *3 or more ADL* | **3.51** | **0.03** | **1.17** | **10.57** |
| GP visits (12 months) | 0.98 | 0.37 | 0.93 | 1.03 |
| Outpatient hospital visits (12 months) | 1.02 | 0.10 | 1.00 | 1.04 |
| Inpatient hospital visits (12 months) | 1.02 | 0.77 | 0.90 | 1.16 |
| Medical card | - | - | - | - |

**note: medical card, ‘yes’ predicts failure perfectly**

Regression results for potentially questionable medications are presented in Supplementary Material Table 4, with statistically significant (p<0.05) associations highlighted bold. There are three such predictors: male (OR 3.39, *p*=.004); three or more ADLs (OR 3.99, *p*=.004); and outpatient hospital visits (12 months) (OR 1.03, *p*=.02).

**Supplementary Material Table 4 Multivariable regression results for potentially questionable medications without imputed values.**

| Variables | Odds Ratio | P>z | [95% Conf. | Interval] |
| --- | --- | --- | --- | --- |
| Gender (male) | **3.39** | **<0.01** | **1.49** | **7.71** |
| Education |  |  |  |  |
| *Secondary* | 0.80 | 0.61 | 0.33 | 1.91 |
| *Third/higher* | 0.69 | 0.51 | 0.23 | 2.06 |
| Age group |  |  |  |  |
| *70-74 years* | 1.77 | 0.64 | 0.16 | 19.72 |
| *75-79 years* | 4.84 | 0.17 | 0.51 | 46.28 |
| *80-84 years* | 1.86 | 0.60 | 0.18 | 19.15 |
| *85 years and over* | 8.57 | 0.06 | 0.93 | 78.64 |
| Cancer | 1.00 | 1.00 | 0.39 | 2.54 |
| Any serious heart condition | 0.73 | 0.49 | 0.30 | 1.79 |
| Any heart risk factors | 1.47 | 0.49 | 0.50 | 4.29 |
| Any serious chronic condition | 1.11 | 0.46 | 0.84 | 1.47 |
| Activities of daily living |  |  |  |  |
| *1 ADL* | 0.49 | 0.38 | 0.10 | 2.39 |
| *2 ADL* | 1.20 | 0.80 | 0.30 | 4.77 |
| *3 or more ADL* | **3.99** | **<0.01** | **1.56** | **10.20** |
| GP visits (12 months) | 0.98 | 0.36 | 0.95 | 1.02 |
| Outpatient hospital visits (12 months) | **1.03** | **0.02** | **1.00** | **1.05** |
| Inpatient hospital visits (12 months) | 0.99 | 0.89 | 0.86 | 1.14 |
| Medical or GP card | 2.00 | 0.52 | 0.24 | 16.86 |

**5) Secondary analysis: gender and specific medications**

Additionally, ad-hoc analyses evaluating prescribing differences between males and females are shown below in Supplementary Material Table 5 and Supplementary Material Table 6.

**Supplementary Material Table 5 prescribing differences between males and females** **for potentially questionable medications.**

|  | N (%) | | | |
| --- | --- | --- | --- | --- |
| Questionable | Female | Male | Total |  |
| Drugs for acid-related disorders, excl. PPI (A02) | 12 (4.9) | 11 (3.9) | 23 (4.4) |  |
| Oral antidiabetics, excluding metformin (A10B) | 6 (2.4) | 7 (2.5) | 13 (2.5) |  |
| Vitamin K antagonists (B01AA) | 22 (8.9) | 35 (12.5) | 57 (10.9) |  |
| Unfractionated heparin (B01AB01) | 0 (0.0) | 0 (0.0) | 0 (0.0) |  |
| Platelet aggregation inhibitors, incl. low-dose aspirin (B01AC) | 113 (45.9) | 156 (55.9) | 269 (51.2) |  |
| Novel oral anticoagulants (B01AE, B01AF) | 5 (2.0) | 8 (2.9) | 13 (2.5) |  |
| Other anticoagulants (B01AD, B01AX) | 2 (0.8) | 5 (1.8) | 7 (1.3) |  |
| Antianemic preparations (B03) | 28 (11.4) | 32 (11.5) | 60 (11.4) |  |
| Blood products (B05A) | 0 (0.0) | 0 (0.0) | 0 (0.0) |  |
| Cardiac glycosides (C01A) | 6 (2.4) | 18 (6.5) | 24 (4.6) |  |
| Alpha-blocker antihypertensives (C02CA, C02LE) | 4 (1.6) | 14 (5.0) | 18 (3.4) |  |
| Low-ceiling diuretics, thiazides & non-thiazides (C03A, C03B) | 13 (5.3) | 9 (3.2) | 22 (4.2) |  |
| Potassium-sparing agents, excl. spironolactone (C03D) | 1 (0.4) | 5 (1.8) | 6 (1.1) |  |
| Non-selective beta-blockers (C07AA) | 6 (2.4) | 5 (1.8) | 11 (2.1) |  |
| Calcium channel blockers (C08) | 49 (19.9) | 54 (19.4) | 103 (19.6) |  |
| Angiotensin-converting-enzyme inhibitors (C09A, C09B) | 51 (20.7) | 69 (24.7) | 120 (22.9) |  |
| Angiotensin II antagonists (C09C, C09D) | 38 (15.4) | 44 (15.8) | 82 (15.6) |  |
| Finasteride (G04CA51) | 0 (0.0) | 0 (0.0) | 0 (0.0) |  |
| Iodine therapy (H03C) | 0 (0.0) | 0 (0.0) | 0 (0.0) |  |
| Antineoplastic drugs (L01) | 3 (1.2) | 4 (1.4) | 7 (1.3) |  |
| Endocrine therapies (L02) | 15 (6.1) | 9 (3.2) | 24 (4.6) |  |
| Immunosuppressants (L04A) | 2 (0.8) | 2 (0.7) | 4 (0.8) |  |
| Anti-gout drugs, excluding allopurinol and colchicine (M04) | 0 (0.0) | 2 (0.7) | 2 (0.4) |  |
| Systemic drugs for obstructive airway diseases (R03C, R03D) | 6 (2.4) | 7 (2.5) | 13 (2.5) |  |
| Any of the above | 175 (71.1) | 226 (81.0) | 401 (76.4) |  |

**Supplementary Material Table 6 prescribing differences between males and females** **for potentially inadequate medications.**

|  | N (%) | | |
| --- | --- | --- | --- |
| Often inadequate | **Female** | **Male** | **Total** |
| Vitamin D (A11CC) | 3 (1.2) | 10 (3.6) | 13 (2.5) |
| Calcium supplement (A12A) | 43 (17.5) | 18 (6.5) | 61 (11.6) |
| Cardiac stimulants other than glycosides (C01C) | 1 (0.4) | 2 (0.7) | 3 (0.6) |
| Antihypertensives, excluding α-blockers (C02) | 0 (0.0) | 0 (0.0) | 0 (0.0) |
| Peripheral vasodilators (C04) | 2 (0.8) | 3 (1.1) | 5 (1.0) |
| Lipid-modifying agents (C10) | 87 (35.4) | 151 (54.1) | 238 (45.3) |
| Immunostimulants (L03A) | 0 (0.0) | 0 (0.0) | 0 (0.0) |
| Bisphosphonates (M05BA) | 17 (6.9) | 3 (1.1) | 20 (3.8) |
| Other osteoporosis drugs (M05B) | 14 (5.7) | 3 (1.1) | 17 (3.2) |
| Antidementia drugs (N06D) | 10 (4.1) | 16 (5.7) | 26 (5.0) |
| Any of the above | 124 (50.4) | 170 (60.9) | 294 (56.0) |

**References**

1. Lee SJ, Lindquist K, Segal MR, Covinsky KE. Development and Validation of a Prognostic Index for 4-Year Mortality in Older Adults. *JAMA.* 2006;295(7):801-808.

2. Kobayashi LC, Jackson SE, Lee SJ, Wardle J, Steptoe A. The development and validation of an index to predict 10-year mortality risk in a longitudinal cohort of older English adults. *Age Ageing.* 2017;46(3):427-432.

3. Matthews S, Ward M, Nolan A, Normand C, Kenny RA, May P. Predicting mortality in The Irish Longitudinal Study on Ageing (TILDA): development of a four-year index and comparison with international measures. *BMC Geriatrics.* 2022;22(1):510.
