# Supplementary material for: Overprescribing among older people near end of life in Ireland: Evidence of prevalence and determinants from The Irish Longitudinal Study on Ageing (TILDA)
Source: PLoS One. 2022 Nov 30;17(11):e0278127. doi: 10.1371/journal.pone.0278127 (PMC9710761; doi:10.1371/journal.pone.0278127)
Supplement: Attachment — Submitted filename: reviewer response.docx [file pone.0278127.s002.docx]

Dear Editor,

Thank you for giving me the opportunity to submit a revised draft of my manuscript. We are grateful to the editors and reviewers for their time and insightful comments on our manuscript. We have addressed their comments and suggestions as appropriate and wish to submit a revised version of the manuscript for further consideration in the journal. Changes in the initial version of the manuscript are recorded using tracked changes. Below, we also provide a point-by-point response explaining how we have responded to each of the comments (changes to text in *red italics*).

On behalf of myself and my co-authors, thank you for agreeing to review our paper, it is very much appreciated We really appreciate the comments that have been made, many of which have helped us to improve our manuscript. We look forward to the outcome of your assessment.

Firstly, we will address your (the editor) comments and concerns below.

**Editor Comments:**

*1.1* *In Objectives, Discussion and other parts of the manuscript, it is stated that the paper describes over prescription in the last year of life. However, this is misleading. In fact, only 13% of the study subjects were in her/his last year of life. This should be amended in the whole text. I would stress the description of the population in the Abstract.* *There is no definition of the inclusion criteria, but a vague sentence about “We identified mortality through the national death registry”.*

**Answer**: Thank you for highlighting the confusion in our text. We have now made some edits that should hopefully clear up any concerns. Our overall sample of 525 participants are those who received a risk score of 11+. Some of whom received potentially questionable (n=294) or potentially inadequate (n=401) medications. These participants are detailed in Tables 1 & 2. We understand that the confusion stems from our Table 3 & 4 analyses that looks at those in the last year of life (LYOL) who receive potentially questionable (n=26) or potentially inadequate (n=42) medications versus those who were not in their last year of life. The regressions that were ran in Table 3 & 4 were on a sample of 525 participants in total – outcome recorded as 0 for participants who were not in LYOL and recorded as 1 if in LYOL (based on prescribing outcome). Therefore, our regressions in Table 3 & 4 are based on LYOL. We have made the following changes on various pages:

Page 3 (line 54): ‘We identified mortality through the national death registry *(died in <365 days versus not)*.’

Page 3 (line 57-59): ‘*Of the 401 participants that had potentially inadequate medications, 42 were in their LYOL. OF the 294 participants that had potentially questionable medications, 26 were in their LYOL.’*

*Page 6 (line 112):* ‘We aimed to describe the prevalence of two undesirable outcomes, and to identify factors associated with these outcomes: potentially questionable prescribing, and potentially inadequate prescribing, in the last year of life *versus not*.’

Page 14 (line 267): ‘Regression results for potentially inadequate medications *in LYOL* are presented in Table 3, with statistically significant (p<0.05) associations highlighted in bold.’

Page 15 (line 273): ‘Regression results for potentially questionable medications *in LYOL* are presented in Table 4, with statistically significant (p<0.05) associations highlighted in bold.’

Page 17 (line 289-292): ‘In our sample, 62% of people in the last year of life *(n = 68 in LYOL)* had potentially inadequate medications, and 38% had potentially questionable medications (Table 2). These figures compare to 79% and 59% respectively among those not in the last year of life *(n = 457 not in LYOL)*.’

To address the inclusion criteria issue we have added the following to the manuscript:

Page 7 (line 141-142): *‘This allows us to identify the TILDA participants from our sample that have died in the last 365 days.’*

Page 9 (line 187-192): ‘*Main inclusion criteria for this study are as follows: a TILDA participant and score of 11+ points on the TILDA mortality index. To identify those in the last year of life, w*e then cross-referenced *potentially inadequate and questionable* prescribing patterns in the CAPI *data* for which the participant first had high mortality risk *against* GRO mortality data *(i.e. died in last 365 days)*. *As mentioned earlier, all deaths in Ireland are recorded with the GRO, and TILDA data are linked to GRO data to March 2018 (27).’*

*1.2 Public health insurance.* *According to Table 1, 34 participants (6,5 %) were no holders of Medical Card. For an international audience, it would be helpful a brief description of the Public health insurance schemes in Ireland and the criteria of residents to apply for them. A very remarkable finding is that 401 out 491 (82%)* *Medical Card holders received potentially inadequate medications, but none of non-holders (Tables 1 and 3). This fact should be developed and explained. I wonder if it suggests any lack of access to prescriptions in no card holders.*

**Answer**: We have now added to the text to further explain the Irish health system and medical cards. The text in Methods on page 10 (line 210) to Page 11 (line 221) now includes: ‘*In Ireland, all persons are technically entitled to receive healthcare through the public health system, but there are variations in costs, coverage and access. An alternative for residents is to pay for private health insurance for access to the public and private healthcare system.(31) A medical card is available to those receiving welfare payments, low learners and retirees and it entitles holders to receive the following free of charge: hospital care, GP visits, dental services, optical and aural services, prescription drugs and medical appliances. Those who received a slightly higher incomes are eligible for a 'GP Visit Card'.* *All people over age 70 and those under 70 meeting a specified low-income threshold have a medical card that subsidises prescription pharmaceuticals. Those without a medical card pay capped co-payments for prescriptions. A small proportion of people over 70 prefer not to take up a medical card as it restricts some of their choice in accessing certain types of health care; this group is wealthier and healthier than the population average(33).’*

We also added the following inserts to the text to clarify the role of medical cards in the sample and in the primary analysis:

Page 12 (line 246-248): *‘Our sample demonstrated that 94% of people have a medical card, reflecting the high age profile of the sample.’*

Page 14 (line 261-263): *‘Our analysis showed that those with a medical card had poor outcomes, reflecting the higher age and higher illness burden of that group, but this does not imply any causal relationship (Table 3).’*

*1.3 As the reviewers highlight, it would be helpful to include further information about the “Mortality Index” and specifically describe the items employed for such score. It seems plausible that the predictors employed are related to morbidity and, consequently, to the need of prescriptions.*

**Answer**: Thank you for highlighting this. Our TILDA mortality index is now published online with BMC Geriatrics and is available as a reference for further details of our index development. We have added the following to the text in the manuscript and the submitted ‘Supplementary file’.

Page 8 (line 151-153): ‘We derived an index following prior work in the HRS family(4, 29); this created a weighted index of demographic, health, functional, risk factors and prior service use that has high predictive power for all-cause mortality (see Supplementary file *for summary based on sample employed in this study*). *The development of our full TILDA mortality risk index is discussed in detail elsewhere (30).’*

Supplementary File Page 1 (line 9-10): ‘*The development of our full TILDA mortality risk index is discussed in detail elsewhere^3^*’

Supplementary File Page 2 (line 9-10): *‘Our final 14-predictor model assigned risk points for: male (1pt); age (65–69: 2pts; 70–74: 4 pts; 75–79: 4pts; 80–84: 6pts; 85 + : 7pts); heart attack (1pt); cancer (3pts); smoked past age 30 (2pts); difficulty walking 100 m (2pts); difficulty using the toilet (3pts); difficulty lifting 10lbs (1pts); poor self-reported health (1pt); and hospital admission in previous year (1pt).’*

*1.4* *The authors count with a database that includes information of 4 different survey waves. I wonder if it would be possible to observe the temporal evolution of prescription patterns.*

**Answer**: The wave-by-wave samples for this analysis are approximately 100 observations each time, too few for a robust time series analysis. While other papers have looked at prescribing patterns in the TILDA sample as a whole over time, we consider that to be beyond the scope of this analysis.

We have added the following to limitations on page 19 (line 352-353): *‘Potentially patterns of prescribing near end of life are changing over time, but the TILDA sample at each wave is too small to analyse rigorously such trends.’*

*1.5 In Methods, it is explained that if follow-up confirmed that a participant in the study had died, a family member or friend was asked to complete an interview (EOLI). If I fully understand it, the information collected in EOLI was not employed in the study, so the reference to EOLI could be avoided.*

**Answer**: I have now removed this line to make the passage read more clearly. We have removed the following on page 7 (line 137-139): *‘From Wave 2, if follow-up confirmed that a TILDA participant had died, a family member or friend was asked to complete a voluntary face-to-face EOL interview (EOLI) about the participant’s LYOL.’*

*1.6 Page 8, line 175:* *the expression “comorbidities” is too ambiguous and needs a better description.*

**Answer**: Thank you for raising this point. We have now added the following bracket description for comorbidities on page 10 (line 203-209): “These include: sociodemographic variables (age, gender and education); health diagnoses (cancer, heart disease, arthritis, comorbidities *(e.g. more than one: heart disease, arrythmia, hypertension, diabetes, lung disease, cancer, Parkinson’s, psychological illness, alcoholism, Alzheimer’s, stomach ulcers or liver disease),* cholesterol, incontinence, cataracts and osteoporosis); functional limitations (activities of daily living, instrumental activities of daily living and vigorous physical activity); prior healthcare use (general practitioner, emergency department, outpatient hospital and inpatient hospital visits in prior 12 months); and public health insurance (medical card and GP visit card).*”*

*1.7 There is a typo in Table 3 (95% Conf. Interval for Males).*

**Answer**: Thank you for highlighting your concern. We have checked the figures and they are correct. If you still feel there is a typo, can you please clarify your concern for us.

*1.8  Please ensure that your manuscript meets PLOS ONE's style requirements, including those for file naming. The PLOS ONE style templates can be found at https://journals.plos.org/plosone/s/file?id=wjVg/PLOSOne_formatting_sample_main_body.pdf and* [*https://journals.plos.org/plosone/s/file?id=ba62/PLOSOne_formatting_sample_title_authors_affiliations.pdf*](https://journals.plos.org/plosone/s/file?id=ba62/PLOSOne_formatting_sample_title_authors_affiliations.pdf)

**Answer**: We have now altered the format of the authors’ names at the beginning of the manuscript to match the template document. We now believe that the manuscript and accompanying materials are formatted correctly. Please let me know if we have made an error.

*1.9 We note that you have provided funding information that is not currently declared in your Funding Statement. However, funding information should not appear in the Acknowledgments section or other areas of your manuscript. We will only publish funding information present in the Funding Statement section of the online submission form. Please remove any funding-related text from the manuscript and let us know how you would like to update your Funding Statement. Currently, your Funding Statement reads as follows: "**This work was funded by the Health Research Board (HRB) in Ireland as part of project # SDAP/2019/012 (PI: May). For more information see www.pelci.ie. The HRB website can be accessed at https://www.hrb.ie/. The funders had no role in study design, data collection and analysis, decision to publish, or preparation of the manuscript. "Please include your amended statements within your cover letter; we will change the online submission form on your behalf.*

**Answer**: My apologies for this error. We have now included the amended statements in the cover letter, as requested. We have now removed funding information from the title page (line 31-32).

*1.10 In your Data Availability statement, you have not specified where the minimal data set underlying the results described in your manuscript can be found. PLOS defines a study's minimal data set as the underlying data used to reach the conclusions drawn in the manuscript and any additional data required to replicate the reported study findings in their entirety. All PLOS journals require that the minimal data set be made fully available. Important: If there are ethical or legal restrictions to sharing your data publicly, please explain these restrictions in detail. Please see our guidelines for more information on what we consider unacceptable restrictions to publicly sharing data: http://journals.plos.org/plosone/s/data-availability#loc-unacceptable-data-access-restrictions. Note that it is not acceptable for the authors to be the sole named individuals responsible for ensuring data access. We will update your Data Availability statement to reflect the information you provide in your cover letter.*

**Answer**: We have now added a full data availability statement to our cover letter. Thank you for updating this for us.

Here is a point-by-point response to the two reviewers’ comments and concerns.

**Reviewer 1 Comments:**

*This study uses TILDA to investigate potential over and under prescribing patterns in the last year of life. The authors identify the cohort (or study sample) using a predictive mortality index, investigate their prescriptions at the time they meet the 'likely to die' threshold, and follow until i) death or ii) for one year. Providing quality end of life care is important, and describing current practice is essential to make improvements. Overall, I found this manuscript a little confusing and the sample was on the smaller side, especially with increasing granularity.*

**Answer**: Thank you for your comments on our manuscript. Our aim is that the edits we have made to the manuscript will improve the understanding of our work. We have specific responses below 2.1, etc.

***2.1*** *It would be helpful for readers if the index used was reproduced in supplementary material. For example, diabetes and lung disease were included in the index, and oral antidiabetics, excluding metformin, and systemic drugs for obstructive airway diseases are included in the questionable drugs classes. So, it seems the cohort chosen may have been more likely to be using a 'questionable drug' (i.e. overestimates inappropriate use).*

**Answer**: Thank you for your comments. Please see the response to comment 1.3 above for further information.

***2.2*** *The interpretation of the OR in section 4.1 appears to be incorrect. I don't believe that either the outcome of potentially inadequate or questionable medications was rare enough to justify approximating OR for RR (in which case, saying 4.4x more likely would be correct). So, something like 'The odds of [men] receiving potentially inadequate medications were 4.4 times higher [than the odds of women receiving potentially inadequate medications], after controlling for other important predictors of prescribing' would be more accurate.*

**Answer**: We have now altered the sentence based on your suggestion on page 17 (line 299-305). We believe it now reads more accurately. The passage now reads: *“The odds of men receiving* potentially inadequate medications *were 4.4 times higher (than the odds of women receiving potentially inadequate medications),* after controlling for other important predictors of prescribing. *Similarly, the odds of men receiving potentially questionable medications were 3.4 times higher (than the odds of women receiving questionable medications), after controlling for the same predictors.”*

***2.3*** *I understand you are using a previous classification, but taken without the context of that study, the terms 'potentially inadequate' and 'potentially questionable' are not as clear as, say, underprescribing or potentially inappropriate prescribing. For example, 'potentially inadequate' could be interpreted as underprescribing (as adequate can be quality or quantity'), but in this case it* *means '[prescribing that has] potentially inadequate clinical benefit' (which qualifies, it means quality). It may be helpful for a global audience to make sure this is clear).*

**Answer**: Thank you for highlighting this. We have now taken your suggestion and added a sentence to add some context. We added the following on page 9 (line 183-186): *“For the purpose of this study, potentially questionable and potentially inadequate prescribing is defined as “prescribing that has potentially questionable/inadequate clinical benefit in terms of quality of care in end-of-life”.”*

***2.******4*** *The paragraph beginning on page 14, line 257 - could you/did you look at the differences in trends between men and women in LYOL vs not? That might be a more interesting inclusion that the current paragraph, which is a little jarring as the literature cited talks about undertreatment of drugs when those same drugs are on the questionable and inadequate lists (i.e. in general, it would be better quality prescribing if people weren't on them at end of life).*

**Answer**: As we already noted in response to 1.4 above, our sample size precludes this kind of secondary analysis. Additionally, we have added the following to the limitations on page 20 (line 354-356): *‘Similarly, temporal comparison of men and women’s patterns before and during LYOL will require larger datasets – and our analyses highlight the need for investigating sex differences in this population.’*

***2.5*** *Do you have any sense as to how well the index worked? For your sample, would prescribers have known they were prescribing at end of life, so that could factor into decisions to continue or cease medicines?*

**Answer**: Our paper presenting the mortality index(1) showed good predictive accuracy with AUC=0.78.

We consider this useful for defining the sampling frame, as we explain in the Methods section with the paragraph starting ‘This sampling frame was applied to narrow down eligibility to those for whom polypharmacy and specific medicines are plausibly inappropriate or questionable.’

At the individual level in real world practice, it seems likely that clinicians are able to detect those who are approaching end of life – hence we see in Table 2 a lower (but still non-negligible) prevalence of potentially inappropriate medications. We believe this is addressed in the first paragraph of 4.1.

***2.6*** *Did you have any information on family support that could be used in the regression?*

**Answer**: Thank you for your question. We have details on whether the person lives alone, with a partner, or with others. There was no association between predictor and outcome, and collinearity between this and other predictors, so it was dropped from the analysis.

**Reviewer 2 Comments:**

***3.1*** *line 175 -* *could you please be more precise about the health diagnoses - ie why is cholesterol per se in the bracket, which comorbidities do you mean etc*

**Answer**: Thank you for raising this point. We have now added the following bracket description for comorbidities on page 10 (line 202-209): “These include: sociodemographic variables (age, gender and education); health diagnoses (cancer, heart disease, arthritis, comorbidities *(e.g. more than one: heart disease, arrythmia, hypertension, diabetes, lung disease, cancer, Parkinson’s, psychological illness, alcoholism, Alzheimer’s, stomach ulcers or liver disease),* cholesterol, incontinence, cataracts and osteoporosis); functional limitations (activities of daily living, instrumental activities of daily living and vigorous physical activity); prior healthcare use (general practitioner, emergency department, outpatient hospital and inpatient hospital visits in prior 12 months); and public health insurance (medical card and GP visit card).*”*

TILDA collects data on 17 serious diseases, and we considered each of these as well as total count of the conditions.

***3.2*** *line 201 - heart risk factor should be defined more precisely in the way it was presented in the legend of Table 1.*

**Answer**: Thank you for highlighting this. We have now incorporated the description from Table 1 so it is defined better in the text. Page 12 (line 242-245) now reads: “Over three quarters of the sample had a heart risk factor *(at least one diagnosis of: hypertension, arrhythmia, high cholesterol)* (82.5%) and more than one *serious* chronic condition (75.8%).

***3.3*** *line 204 - did the patients have more than one serious condition or more than one health condition. There seem to be a discrepancy between the text and the table (1).*

**Answer**: We have now corrected the discrepancy within the text so it matches Table 1. Same paragraph as is highlighted in the above response to comment 3.2.

**Reference**

1.	Matthews S, Ward M, Nolan A, Normand C, Kenny RA, May P. Predicting mortality in The Irish Longitudinal Study on Ageing (TILDA): development of a four-year index and comparison with international measures. BMC Geriatrics. 2022;22(1):510.
