# Supplementary material for: Overprescribing among older people near end of life in Ireland: Evidence of prevalence and determinants from The Irish Longitudinal Study on Ageing (TILDA)
Source: PLoS One. 2022 Nov 30;17(11):e0278127. doi: 10.1371/journal.pone.0278127 (PMC9710761; doi:10.1371/journal.pone.0278127)
Supplement: Attachment — Submitted filename: Response to Reviewers.docx [file pone.0278127.s003.docx]

The authors have been responsive to several of the previous comments. However, I consider that some major points need to be addressed.

1.- Population of study:

- If I have interpreted the manuscript correctly, the population of study consists in 525 individuals approaching end of life (score of 11+ points on the TILDA mortality index). If my inference is correct, the text following line 186 is misleading. Were the two outcome variables coded as 1 exclusively in participants when their reported medications meet the defined criteria AND they died within 365 days of the CAPI? Please clarify.

>Thanks, you’re right, we have deleted extraneous material and now this section focuses on how the outcome is defined (line 186+ in the old manuscript, line 166+ now).

- Moreover, revise the objectives (line 107 and following). This section includes two expressions that are close, but different. The first sentence states: “We aimed to describe… in the last year of life versus not”, but the second one: “This analysis quantifies … among older people with high mortality risk in Ireland”

> I have standardised to ‘describe’ in both.

- Lines 58, 60 and 267. If my interpretation is correct, the expression “LYOL” should be replaced by “approaching end of life” or another similar.

> LYOL is the more specifically correct because we have defined the outcome as having these prescribing patterns in the last 365 days of life. However, approaching end of life carries the same meaning in this context and we are happy for you to make this change at editor discretion.

- Besides, I will suggest a modification to the first paragraph of Discussion section. Usually, the first sentence summarizes the major findings related to the objectives of the study. The comparison of percentages in people in their last year of life and not is of interest and could be included later in this section, but not in the first sentence. It does not provide a clear answer to the objectives of the study.

>We have restated our main findings from the abstract in the first paragraph of the Discussion.

2.- Public health insurance

- According to the manuscript, the inclusion in the Medical Card Program is associated with potentially questionable medications. However, such association is even more pronounced for inadequate medications, since all of the individuals receiving inadequate medications were Medical Card holders and none of the no-holders received inadequate medications. I agree that it is not possible to estimate an OR when one group includes zero subjects, but the association between Medical Card and inadequate medications has to be recognized and developed.
- Besides, the relationship between Medical Card and potentially inadequate and questionable medications needs further explanation in the Discussion section. Did the no-holders receive a better attention due to their lack of questionable medications? Otherwise, does this finding suggest any lack of access to prescriptions in no card holders? Please, provide plausible hypotheses for this fact.

>There are several plausible hypotheses which may explain this finding. Non-holders are those with higher income levels, and hence they may have a lower propensity for co-morbidities and conditions severity, potentially leading to lower levels of treatment. Free GP visits and access to medication with a small prescription charge for may also make Medical Card holders more likely to be prescribed medications, and specifically inadequate or questionable medications. Discussion extended lines 303+.

Minor comment

- Line 158: TILDA EOLI is not included in the manuscript, so this sentence may be difficult to be interpreted.

>Thanks, good point, we have revised our explanation of what this is.
